# Use of electronic health records in the design and evaluation of implementation research in primary care - experiences from the aspire cluster randomised controlled trials (CRCTS)

**DOI:** 10.1186/1745-6215-16-S2-P33

**Published:** 2015-11-16

**Authors:** Peter Heudtlass, Robbie Foy, Liz Glidewell, Suzanne Hartley, John Turgoose, Tom Willis, Amanda Farrin

**Affiliations:** 1 University of Leeds, Leeds, West Yorkshire, UK

## 

The use of routine datasets in the evaluation of interventions in clinical trials is increasing. Action to Support Practices Implementing Research Evidence (ASPIRE) is an NIHR Programme aiming to develop and test ways to support general practices in implementing key NICE recommendations. Multifaceted intervention packages have been developed to target four key clinical practice recommendations, which will then be evaluated in two parallel cluster randomised controlled trials (cRCTs) in 144 general practices across West Yorkshire.

Practice level data on adherence to recommendations were required to inform the sample size calculation and will also be used to assess the effectiveness of the intervention packages. Data on the number of patients within a practice managed according to the targeted recommendations are obtained remotely, at regular intervals, by a central trials team and are based on Electronic Health Records (EHR) from SystmOne.

Randomisation took place in April 2015 and the intervention packages are currently being implemented. We will present our experience in the use of EHR in the design and proposed analysis. This will include the development of queries to correspond to the clinical recommendations and the methodology used to extract and clean the data from GP based EHR.

We have demonstrated that the use of large routine datasets to inform the design and primary endpoint analysis, whilst ensuring that the required standards of clinical trials are maintained, is achievable. We believe our approach can be used to inform future RCTs, particularly in those in primary care or implementation science.

